# 

**Published:** 1872-01

**Authors:** 


### INDEX.

**A** PAGE

Adams' Class Valedictory.............. 5
Anæsthesia—*Hay*....................... 28
At Last................................ 116
American Med. Association.....116, 255, 302
Anæmic Murmurs, Diagnosis........... 347
Aphasia, Amnesic and Ataxic........... 352
Ani, Prolapsus......................... 364
Abortion, Med., Act to prevent Sale..... 366
Ammonia Injection, Chloroform........ 383
Asthma, Treatment................. 424, 760
"American Plan"....................... 447
Antiphlogistic Treatment of Disease.... 474
Atmosphere, Nervous.................. 504
Albumen, Cold Alcoholic Test ......... 508
Amaurosis, Tobacco.................... 618
Allen, Introductory R. M. C............ 683
Artificial Feeding of Infants, Pepsin .... 701

**B**

Book Notices. ... { 40, 114, 177, 238, 317, 378 / 441, 497, 571, 633, 680, 757 }
Bromide Calcium, Nervine.............. 58
Berlin and Vienna Med. Schools........ 96
Blennorrhagia, Treatment.............. 104
Beef Essence and Potash Salts.......... 141
Belladonna, Action of................... 160
Bright's Disease, Ophthalmoscope...... 220
Buenos Ayres, Plague.................. 235
"Boast Not"........................... 384
Blood Stains........................... 490
Brainard Medical Society............... 570
Baths in Small Pox..................... 698

**C**

Commencement Twenty-ninth, Rush Medical College..................... 1
Characteristic Slur..................... 43
Clinical Lecture—*Barnes*............... 52
Cutaneous Affections, Symmetry....... 59
Carbol. Acid and Sulphite in Small Pox.. 61
Clinical Lecture—*Hutchinson* .......... 78
Clavicle, Fracture—*Sayre*.............. 103
Celibacy and Marriage.................. 127
Cerebro-Spinal Meningitis— 186, 226, 321, 538, 640
Cæsarean Operation in U. S............ 210
Cancrum Oris......................213, 382
Corns, Curiosities of.................... 253
"Cure," Proper Definition—*Eve*........ 296
Chloroform—Ammonia ................ 383
Chinese Surgery........................ 446
Chicago Relief Fund.................... 500
Carbuncles, Treatment.................. 506
Central Illinois Medical Society......... 511
Chloral Hydrate, Lecture—*Etheridge*.. 521
Cholera................................. 601
Cataract, History of Linear Extraction.. 660

PAGE

College Courant......................... 693
Correspondence—*Proegler*.............. 744
Carbolic Acid in Scarlatina.............. 764
Cholera Remedy......................... 764

**D**

Disinfectants.........................64, 143
Death, Signs of......................89, 197
Derivatives, Action of................... 94
Disease Germs—*Danforth*...........167, 449
Diplomas, Bogus........................ 180
Dyspepsia of Liquids.................... 212
Discharges, Uterine and Vaginal........ 291
Dyspepsia, Nux Vomica................. 331
Dyspepsia, Myrrh....................... 383
Disease, Antiphlo. Treatment.......... 474
Disease, Modern Idea................... 505
Drains, Smells and Fevers.............. 639
Dyspepsia, Citrate Iron and Bismuth... 699
Dyspepsia, Arsenic in................... 763

**E**

Etheridge, Prof., Valedictory........... 10
Eye and Ear Infirmary.................. 45
Eczema, Cure of.....................47, 121
Ear, Clinical Lecture—*Holmes*....65, 275, 454
Enuresis............................... 233
Eye and Menstrual Disorders........... 361
Epulis, Malignant, Operation —*Freer* .. 402
Evidence, Medical...................... 504
Electrolysis in Ovarian Tumors......... 508
Ears vs. Brains......................... 510
Electro-Therapeutics—*Peck*............ 588
Eye, Lecture—*Holmes*.................. 596
Electrical Instruments for Medical Use.. 665
Erysipelas ............................. 704

**F**

Femur, Oblique Fracture—*Owens*...... 69
Fever, Treatment....................... 150
Fœtation, Extra Uterine................ 155
Foreign Correspondence—*Tombocken*.... 193
Fractures, Bavarian Method............ 251
Faber's Talking Machine............... 437
Fruit in Rooms......................... 488
Feeding per Rectum.................... 507
Feeble Minded Children, Circular....... 512
Felon, Conseq. and Treat.—*Proegler*, 527, 656
Floral Guide for 1873, Vick's ............ 763

**G**

Graduates, Rush Med. Col., 1871–2..... 1
Gelatinæ Medicatæ..................... 45
Grotto Cure—*Kossuth*.................. 126
Gelseminum in Irritable Bladder. ...... 447
Gunshot Wound of Stomach and Kidney —Recovery—*Brooks*................ 519
Gases vs. Contagion..................... 530

**H** PAGE

Hyperæsthesia—*Hay* 28
Homœopathy, Spiritualism 44
Holmes, Clinical Lecture, Ear 65
Hemorrhoids in Pregnant and Puerperal Women 83
Hospital, the Modern 116
Hypodermic use of Cor.Sub. in Syphilis 162
Hydrate of Chloral, Externally 191
Hernia, Reduction, New Method 192
Hospital Cases, St. Luke's—*Owens* 278
Heat as a Poison 382
Hemorrhagic Variola 401
Heart, Disease of Muscular Walls 425
Hernia, Strangulated 434
Hydrophobia 557
Hysteria 562

**I**

Introductory—*Hay* 28
Infection, Purulent 102
Items, News and Gossip... 117, 184, 240, 443
Insanity, Increase of 121
Impotency 121, 440
Insanity, Responsibility 183
Infantile Paralysis and Deformities 205
India, Women in 252
India Rubber, How Collected 253
Iliac Tumor, Operation—*Prof. Freer* 281
Intussusception, Novel Treatment 288
Illinois State Medical Society 320
Indiana State Medical Society 367
Ivy Poisoning 384
Items—*Etheridge* 717

**J**

Jurisprudence—Confession no Proof of Guilt 702

**K**

Kidney, Gunshot Wound—Recovery 519
Knee Joint, Lateral Dislocation—Recov. 520

**L**

Lime, Syrup Lacto-Phosphate 128
Lightning Stroke 254
Labor, Determining Cause 339
Library, National Medical 637
Leucocythæmia, Pathol. of—*Danforth* 705

**M**

Military Tract Med. Association 22, 407
Microscopy in Syphilis 100
Milk, Artificial 123
Marriage vs. Celibacy 127
Methylene, Bichlorate 128
Malingering Extraordinary 232
Menstrual Disorders and Eye 361
Membranous Form. on Mucous Surfaces. 545
Meteorology, Utility, Instruments 550
Monstrosity—*Brooks* 593
Medical Progress and News—*Etheridge*. 641
Mortality Statistics of the Professions 695

**N**

Nails, Growth of, in Diagnosis 344
"Notes from the Rear Rank"—*Newkirk* 457
Nostrum—(Editorial) 499
Nervous Atmosphere 504
Nose, Removal of Foreign Body 703
Neuralgia, 2nd Lecture—*Hay* 724

**O**

Ophthalmia, Neonatorum, Treatment 76
Obituary, Prof. S. H. Dickson 320
Obstetrics, American Journal 320
Ovarian Tumors, Electrolysis 508
Ovariotomy, (Case)—*Owens* 513

**P** PAGE

Phthisis and Tuberculosis, Acute 78
Purulent Infection 102
Pamphlet Notices—
115, 178, 238, 319, 379, 442, 498, 635, 682, 759
Practice, Sale of, Legal Opinion 180
Penis, Erroneous Constrictions 204
Philos.of Medicine, Intro. Lect.—*O'Brien* 257
Puerperal Convulsions 335
Paralysis, Cerebral, Nails 344
Prolapsus Ani 364
Pyæmia, Experiments 409
Pruritus Vulvæ, Treatment 446
Prize Essays 510
Panaritium—*Proegler* 527, 656
Poisoned Wound—*Paoli* 595
Phosphorus in Skin Diseases 700
Pepsin 701
Placental Respiration, Artificial—*Hyde* 743
Parturition not necessarily a Painful Process—*Wilson* 746

**Q**

Quinine, Uses and Abuses—*Manson* 106

**R**

Rush Medical College 1, 380, 638, 683
Rheumatism, Acute, Prophylactic 49
Rectum, Feeding by 507
Retroversion of Unimpregnated Womb 577
Recollections of a German Surgeon 605
Register and Directory, Chicago. 635, 694, 757
Review Medical Progress and News 641

**S**

Sulphites, Marsh Fevers 50
Small Pox, Carbolic Acid, etc. 61, 178, 250
Shoulder Joint Excisions 62
Syphilis, Microscopic Diagnosis 100
Spleen, Spontaneous Rupture 120
Sponge Tents—*Adolphus* 171
Surgical Case, Remarkable 281
Spermatorrhœa, Treatment 299
"Soothing Syrup," Poison 358
Syphilitic Lesions of Viscera 385
Small Pox, Vaccination and Re-Vac 418
Sanitary Reform, (Editorial) 573
St. Joseph's Hospital 575
"Slaughter of the Innocents" 696
Styptic Cotton 703
Spinal Nerves, Exposure, etc.—*Freer* 712

**T**

Therapeutics of Present Day 57
Transfusion 58, 129, 249
Thermometry, Clinical 445
Tumors, Michel's Process of Removal 509
Tobacco Amaurosis 618
Tuberculous Matter, Inoculation 704
Therapeutical Notes—*Elder* 737

**U**

Uteri Sclerosis, Clinical Lect 71
Uterus, New Action 124
Uræmia, (Case)—*Ford* 287
Uterine and Vaginal Discharges 291
Uterus, Catarrh of—*Hennig* 460
Uterine Tumor, Large, Removal 489
Ulcer of Rectum and Anus—*Owens* 733

**V**

Vaccination Report 189
Vaginal Tumor Removed—*Jackson* 283
Voice—Faber's Talking Machine 437

**W**

Women, Disease, Local Exam.—*Barnes*. 5[illegible]

**PARTICULAR NOTICE**
☞ ***THE JOURNAL will not be sent, after the present number, to any who have not renewed their subscriptions.***

☞ **NOTICE.—During the great fire our subscription book was burned. Subscribers will please send us their names at earliest moment.**
☞ **Remit $3 for the new volume, commencing January, 1872.**

